# Low Resilience of the Particle-Attached Bacterial Community in Response to Frequent Wind-Wave Disturbance in Freshwater Mesocosms

**DOI:** 10.1264/jsme2.ME13032

**Published:** 2013-12-13

**Authors:** Keqiang Shao, Guang Gao, Xiangming Tang, Yongping Wang, Lei Zhang, Boqiang Qin

**Affiliations:** 1 State Key Laboratory of Lake Science and Environment, Nanjing Institute of Geography and Limnology, Chinese Academy of Sciences, 73 Beijing East Road, Nanjing 210008, China; 2 Graduate School of Chinese Academy of Sciences, Beijing 100039, China; 3 State Key Laboratory of Hydrology-Water Resources and Hydraulic Engineering, Nanjing, 223 Guangzhou Road, Nanjing 210029, China

**Keywords:** shallow lake, particle-attached bacterial community, wind-wave disturbance, resilience, resistance

## Abstract

The most common natural disturbances in shallow lakes are wind-induced waves, which cause catastrophic changes in the aquatic fauna of lakes. Recovery from these changes is always prolonged. The objective of this study was to understand the resilience and recovery of the particle-attached bacterial community composition (PABCC) after frequent wind-wave disturbance in a large shallow eutrophic lake. To accomplish this, we designed a mesocosm experiment including an undisturbed control, and a physically disturbed treatment that stimulated the superposition of two different intensities of wind-induced waves in the large shallow eutrophic Lake Taihu, China. The PABCC was determined by denaturing gradient gel electrophoresis, following by cloning and sequencing of the selected samples. We observed that the most marked change of the PABCC occurred in the disturbed treatment, in which the concentrations of suspended solids (SS) and the water turbidity varied strongly. However, we observed low recovery of the PABCC within 4 days post-disturbance when the investigated environmental factors had also recovered. Our results indicated that the resistance of the PABCC is low, and resilience is also low following frequent disturbance by wind-waves in a large shallow eutrophic lake.

The bacterial community plays a key role in microbial food webs and in the cycling of major elements in aquatic ecosystems. Most natural environments are subject to fluctuations over time (39), and understanding how their bacterial community responds to such disturbances will contribute to predicting the responses of ecosystem processes to environmental changes, based on the bacterial community composition (BCC) (2).

Although the topic of disturbance responses of BCC is attracting more attention, few studies have produced consistent results. Some studies have shown that disturbed microbial communities cannot recover or that recovery is slow (6, 7). Other research has suggested that the responses of aquatic microbial communities showed low resistance but rapid resilience after perturbation (30), as after a sudden storm-mediated disturbance (14, 39).

Disturbance is an important structuring event for freshwater bacterial dynamics (30). In shallow lakes, wind-induced waves are among the most important and natural disturbances. This type of disturbance generally occurs over temporal scales from minutes to hours (20, 34), especially in many large shallow lakes (38), which causes catastrophic changes in the aquatic fauna of lakes. Recovery from these changes is always prolonged. However, little is known about the recovery of the particle-attached bacterial community composition (PABCC) after frequent wind-wave disturbance, especially in a large shallow lake. Unlike other disturbance experiments focusing on one or two specific factors, the pulse effect of wind-induced waves on shallow lake ecosystems represents the superposition of different intensities of disturbance. Of particular interest for the study of wind-induced wave processes is Lake Taihu in eastern China. This eutrophic lake, lying in the delta of the Yangtze River, is the country’s third largest (2238 km^2^) and shallowest (maximum water depth<3.3 m, mean depth=1.9 m), freshwater lake (27). Wind-induced wave processes frequently occur in the lake, due to its shallowness and particular shape (41). In the current study, we thus analyzed the PABCC before, during and after frequent disturbances that simulated wind-induced wave processes. We designed a mesocosm experiment with 2 treatments × 3 replicates × 9 time points, including a physically disturbed treatment with two different degrees of disturbance and an undisturbed control. We set turbidity as the criterion of disturbance, and used different degrees of turbidity to simulate different wind-induced wave processes in Lake Taihu according to Fan *et al.* (9). The PABCC was assessed with polymerase chain reaction-denaturing gradient gel electrophoresis (PCR-DGGE) of the 16S rRNA genes, followed by cloning and sequencing of the selected samples. We observed low resistance and low resilience of the particle-attached bacterial community during frequent wind-wave disturbance.

## Materials and Methods

### Experimental set-up

In the Taihu Laboratory for Lake Ecosystem Research (TLLER, 31°24′-N, 120°13′-E), a mesocosm experiment was conducted for 20 continuous days, between 1st and 20th December 2009. Sediments and water used for the experiment were obtained from Meiliang Bay in Lake Taihu. Six cube-shaped glass containers (50 cm×40 cm×30 cm, only open at the top) were placed in a 10 cm deep layer of sediment with 20 L of overlying water that was pre-filtered through a 64-μm pore net for two day before the experiment.

We set turbidity as the criterion of disturbance and used different degrees of turbidity to simulate different wind-induced wave processes in Lake Taihu according to Fan *et al.* (9). The six containers were set up as two treatments, control (C) and disturbance (D), with each treatment having three containers. The C treatment did not receive any disturbance. In the D treatment, the first 6 days were set as low-intensity disturbance (LID, turbidity: 15.64±8.57 NTU), simulating 3–5 m/s wind-induced waves. Days 7–10 did not receive any disturbance. Days 11–16 were set as high-intensity disturbance (HID, turbidity: 106.87±15.67 NTU), simulating >8 m/s wind-induced waves. Days 17–20 did not receive any disturbance. The disturbance mode of LID or HID was treated with a disturbance/pause–cycle of 4 h/8 h each day, which stimulated natural hydrodynamic processes in the shallow lake.

### Sampling and physicochemical analysis

Water was sampled on days 0, 1, 3, 6, 10, 11, 13, 16, 20. On each sampling day, we sample 500 mL water at 8:00 every morning, 1 h before disturbance. Water for analysis of the PABCC (250 mL) and physicochemical analysis (250 mL) was kept in separate sterile bottles. All water samples were stored at 4C until further analysis. During the experiment, water temperature (T), and turbidity (NTU) were measured using a Multi-Parameter Water Quality probe (YSI 6600). Concentrations of total nitrogen (TN), total phosphorus (TP), and suspended solids (SS) were analyzed in the laboratory according to the previous method (13).

### Bacterial biomass collection, Total bacterial abundance, DNA extraction, and PCR-DGGE

About 250 mL water samples were pre-filtered through a 5 μm pore polycarbonate filter membrane (47-mm diameter; Millipore, Bedford, MA, USA) to collect particle-attached bacterial biomass (19). The abundance of bacteria was determined by epifluorescence microscopy after 4′,6-diamidino-2-phenylindole (DAPI) staining (25) as described previously (35). The filters were stored at −80C until DNA extraction. Bacterial genomic DNA was extracted from the filters as described previously (33).

Mixed DNA from triplicate extractions was used as PCR templates. We used the mixed DNA from the samples collected from the three replicates as the PCR template in subsequent DGGE analysis. The bacterial 16S rRNA gene fragments were PCR amplified using the universal bacterial primer F341 (5′-CCTACGG GAGGCAGCAG-3′) with a GC-clamp attached to its 5′ end and R518 (5′-ATTACCGCGGCTGCTGG-3′) (22). Then, three parallel PCR reactions were performed and pooled for each sample. The sizes of PCR products were verified on a 1.2% agarose gel. The same amount of mixed PCR products for all samples was loaded onto 8% (w/v) polyacrylamide gels (acrylamide and N, N′-methylene bisacrylamide at a ratio of 37.5:1) with a denaturing gradient that ranged from 30 to 50% (where 100% is defined as 7 M urea and 40% deionized formamide). Electrophoresis was performed with a DGGE-2001 system (CBS Scientific, Del Mar, CA, USA). The gel was run initially at 20 V for 15 min and then at 100 V for 16 h at 60°C in 1×TAE running buffer. The gels were stained with SYBR Green I solution (1:10 000 dilution; Amresco, Solon, OH, USA) for 30 min and photographed with an Omega 10 gel documentation system (Ultra-Lum, Claremont, CA, USA). The DGGE banding patterns were analyzed with a gel documentation system, GelCompar II software (Applied Maths, Austin, TX, USA).

### Cloning and sequencing of selected samples

Before cloning and sequencing, we examined the abundance of particle-attached bacteria and the Bray-Curtis similarity of the community. The results indicated that the bacterial community of the control treatment from days 0 to 20 was very similar, and so we used DNA from the disturbed treatment in later cloning and sequencing analysis. Therefore, only 3 clone libraries were generated from the obtained bacterial 16S DNA templates on days 0, 10 and 20 of D treatment to analyze the recovery of PABCC following frequent wind-wave disturbance. The bacterial 16S rRNA genes for sequence analysis were PCR amplified using the eubacterial forward primer 27F (5′-AGAGTTTGATCMTGGCTCAG-3′) and the universal reverse primer 1492R (5′-GGTTACCTTGTTACGACTT-3′) (23). For each sample, products of three parallel PCRs were mixed. The mixed PCR products were purified immediately with the E.Z.N.A. Cycle-Pure Kit (Omega Bio-Tek, Norcross, GA, USA) and cloned into pMD18-T vector (Takara Bio, Otsu, Japan), then transformed into competent *Escherichia coli* DH5α cells according to the manufacturer’s instructions. Subsequent to verification by PCR amplification using vector primers, the positive clones were randomly selected for sequencing, which was performed on an ABI PRISM 3730 automated DNA capillary sequencer (Applied Biosystems, Foster City, CA, USA).

Raw sequence data were processed and checked with BioEdit software. All sequences obtained were checked for potential chimeric sequences using the CHECK_CHIMERA program. The remaining sequences were clustered into operational taxonomic units (OTUs) with a 0.03 cut-off value using the “cluster” command in the Mothur software (29).

### Statistical analysis

Based on the PCR-DGGE results, the Bray-Curtis distance between the communities before (Day 0) and after the disturbance (day 1, 3, 6, 10, 11, 13, 16 and 20) in each of the two treatments was used to estimate the particle-attached bacterial community resistance (30). Here, the PABCC on day 0 in the C and D treatments (C0 and D0, the mixed DNA from the three samples at day 0) was used as a reference, respectively. For each treatment, we calculated the difference in similarity between day 0 and every other day. To summarize the overall recovery at the end of the LID or HID, the Bray-Curtis similarity of D treatment from day 10 or 20 to day 0 was compared using PRIMER 5 software (http://www.primer-e.com/).

The slopes of linear regression analyses between all pairwise Bray-Curtis similarity indices and the observation days were calculated to determine the particle-attached bacterial community rate of change (resilience) (30) in D treatment after wind-wave disturbance. The LIBSHUFF program was used to statistically assess the differences among three clone libraries (31).

### Nucleotide Sequence Accession Numbers

The accession numbers of the sequences obtained in this study and deposited in GenBank were JN705812–JN705913.

## Results

### Changes of physicochemical parameters

**Fig. 1:**
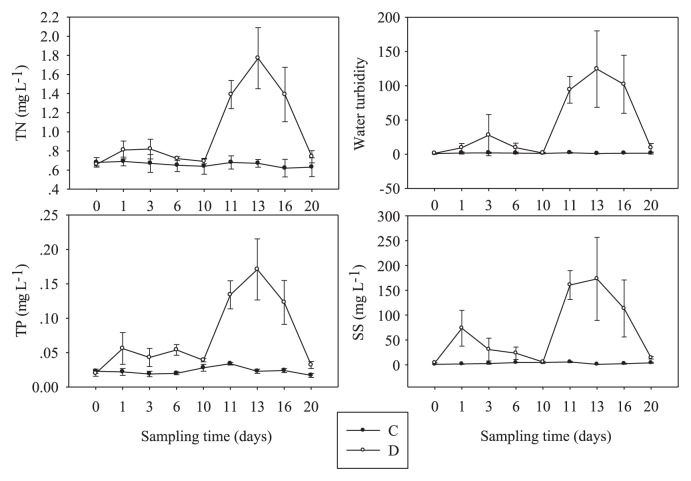
Temporal changes in physicochemical parameters in the water column during the disturbed treatment (D, open circles) and untreated control (C, solid circles). Data are the means and standard deviations of the 3 replicates, TN, total nitrogen; TP, total phosphorous; SS, suspended solids.

The temporal changes in 4 measured physicochemical parameters in the water column of C and D treatments are shown in Fig. 1. At the beginning and end of disturbance (LID or HID) during this experiment, the measured environmental variables were similar in D treatment. At other times, these variables differed (Fig. 1). The water temperature during the experiment ranged from 17 to 20°C. In the control treatment, the concentrations of TN, TP, SS and turbidity generally remained stable over the 20-day period of the experiment. In contrast, in the disturbed treatment, the concentrations of TN, TP, SS, and turbidity varied and increased significantly with the development of disturbance and then returned to baseline levels within 4 days of no disturbance. Furthermore, the higher disturbance (HID) resulted in significantly higher concentrations of TN, TP, and SS, and turbidity than did the lower disturbance (LID).

### Changes of the particle-attached bacterial abundance

**Fig. 2:**
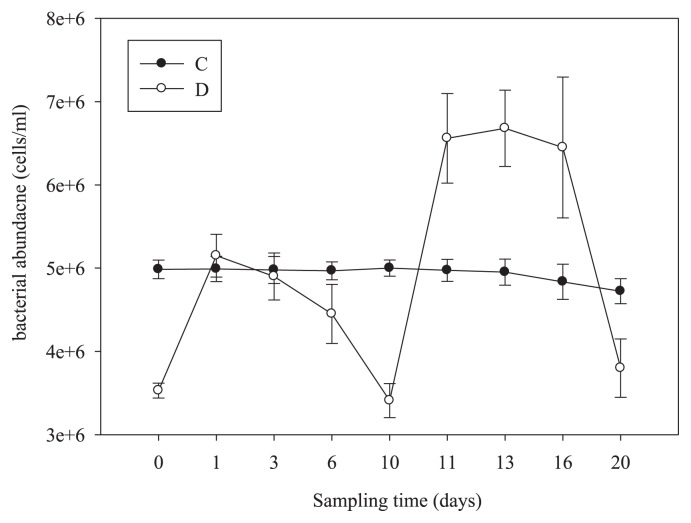
Temporal changes of particle-attached bacterial abundance in the disturbed treatment (D, open circles) and untreated control (C, solid circles). Data are the means and standard deviations of the 3 replicates.

Fig. 2 shows that the temporal changes of particle-attached bacterial abundance in C and D treatments. In the control, the abundance of particle-attached bacteria generally remained stable for 13 days, then decreased slightly with time. In contrast, in the disturbed treatment, the abundance of particle-attached bacteria varied and increased significantly with the development of disturbance and then returned to baseline levels within 4 days of no disturbance. Furthermore, higher disturbance (HID) resulted in significantly higher abundance of particle-attached bacteria than lower disturbance (LID).

### DGGE analysis of the particle-attached bacterial community

The changes in the bacterial community structure of the 18 samples of C and D treatments, evaluated by DGGE analysis, are shown in Fig. S1. In the control, the DGGE profiles of bacterial DNA extracted from enclosures showed highly similar patterns for 13 days, and then the number of recognized DGGE bands slightly changed with time. In the disturbed treatment, from days 1 to 6, there was little variation in the PABCC compared to that observed on day 0; from days 11 to 16, there was much variation in the PABCC compared to that observed on day 0. On day 10, the PABCC showed patterns similar to those observed on day 0, and on day 20, the PABCC showed patterns different to those observed on day 0 (Fig. S1).

To measure the resistance of particle-attached bacterial communities to different level of disturbances, we compared the average and minimum daily Bray-Curtis values obtained before (Day 0) and after disturbance in C and D treatments (Table 1). The results showed that the particle-attached bacterial communities varied immediately after disturbance, but did not change from days 0 to 13 in the control treatment, and then slightly changed. The bacterial community of the control treatment from days 0 to 20 was very similar. However, the communities in the control treatment showed the highest average and minimum daily Bray-Curtis similarity, and the communities in the LID were slightly resistant, showing lower average and minimum daily Bray-Curtis similarity, whereas the HID was least resistant. This disturbance showed the lowest average and minimum daily Bray-Curtis similarity (Table 1).

**Fig. 3:**
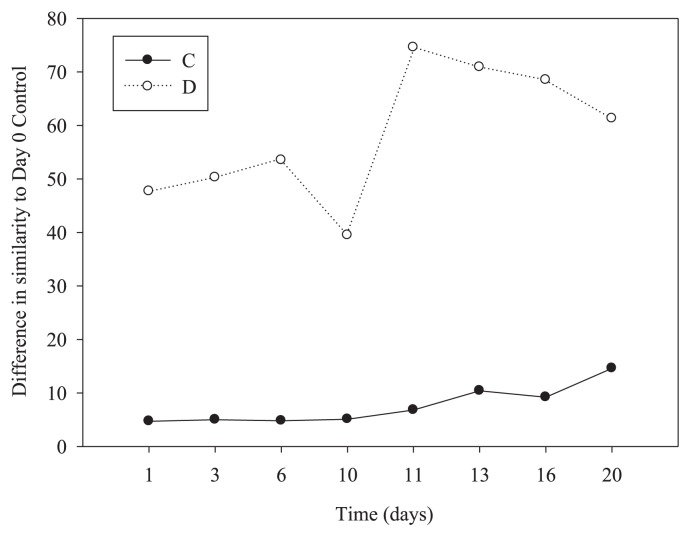
Recovery was calculated as the difference in Bray-Curtis similarity determined from PCR-DGGE results for the disturbed treatment (D, open circles) and untreated control (C, solid circles) to day 0.

Although the resistance of particle-attached bacterial communities with these two disturbances was low, the recovery of LID was relatively strong and rapid, whereas the recovery of HID was slight and slow (Table 1; Fig. 3). The community in the control after day 20 was most similar to the community at day 0 (85.4% similarity), suggesting that all of the communities ultimately showed no change by the end of experiment (Table 1; Fig. 3). The community in the disturbed treatment within 4 days after the end of LID (day 10) was more similar to the community on day 0 (60.5% similarity) than during disturbance (days 1 through 6), suggesting that all of the communities ultimately achieved a degree of recovery by the end of LID. The community in the disturbed treatment within 4 days after the end of HID (day 20) was less similar to the community on day 0 (only 38.7% similarity) than during disturbance (days 11 through 16), suggesting that all of the communities ultimately achieved slight recovery by the end of the experiment (HID) (Table 1; Fig. 3).

**Fig. 4:**
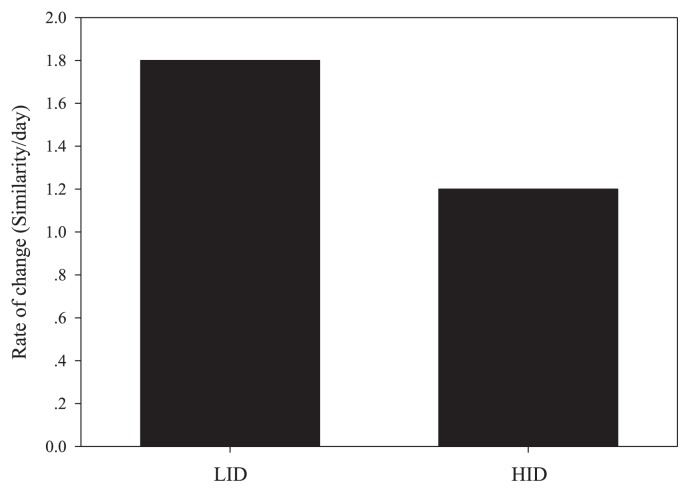
Particle-attached bacterial communities change rates (resilience), as calculated by the slope of the Bray-Curtis similarities determined on PCR-DGGE profile over time between observations in the disturbed treatment. LID, low-intensity disturbance; HID, high-intensity disturbance.

Linear regression analysis revealed that the rate of change, or resilience, of the bacterial community during LID was relatively rapid, approximately 0.5 times higher than that during HID (Fig. 4), suggesting that the resilience of the PABCC is low following frequent disturbance.

### Clone Library Coverage and Diversity indices, LIBSHFF analysis

The results of the bacterial abundance and the Bray-Curtis similarity indicated that the bacterial community did not change from days 0 to 13 in the control treatment, and then slightly changed. The bacterial community of the control treatment from days 0 to 20 was very similar. Therefore, we only constructed a clone library for D treatment. A total of 255 16S rRNA sequences (85 prior to disturbance, 85 after LID and 85 after HID) in the disturbed treatment were analyzed to identify the PABCC (Table 2). These sequences contained 102 OTUs. The samples from the disturbed treatment on day 10 and day 20 contained the most OTUs (50 and 31, respectively), whereas the sample from the disturbed treatment on day 0 contained the fewest (21). A comparison of the observed number of OTUs revealed that library D10 had lower coverage value (63.5%), whereas libraries D0 and D20 had the highest (89.4% and 76.5%, respectively). This comparison indicated that the D10 sample had higher diversity, whereas D0 and D20 samples had the lowest diversity (Shannon indices were 3.64, 2.42 and 2.62, respectively) (Table 2).

The LIBSHUFF program was used to determine if the clone libraries from the D0, D10, and D20 samples were drawn from the same populations in the disturbed treatment. Results of statistical comparison showed that the day-20 clone library and day-0 clone library (day-10 clone library) were significantly different from each other, as indicated by the lower *p* value of <0.001 (31). However, there were no significant differences between the day-0 clone library and the day-10 clone library (Table 3).

## Discussion

The aim of this study was to understand the resilience and recovery of the PABCC after frequent disturbance that simulated two different intensities of wind-wave processes in a large shallow lake. The PABCC before, during and after the disturbance were assessed by PCR-DGGE of the 16S rRNA genes, followed by cloning and sequencing of the selected samples.

In shallow lakes, sediment re-suspension is often a direct result of wind-wave disturbance (1) and contributes to a marked extent to the total settling flux (8, 28). A previous study reported that in the lake center of Lake Taihu, moderate wind (3.3 to 5.0 m s^−1^) could cause strong mixing of the water column and sediment re-suspension during almost two-thirds of the year (40). Sediment re-suspension creates a pulse of nutrients trapped in the surface layer of the bottom sediments fluxing into the water column. The disturbance-induced increase in physicochemical parameters in the water column that we measured agreed with that reported in numerous previous studies (10, 21).

Sediment re-suspension can be an important mechanism for the exchange of organic matter and attached bacteria between sediments and overlying water (12). In our study, the results of the bacterial abundance and the Bray-Curtis similarity indicated that the bacterial community did not change from days 0 to 13 in the control treatment, and then slightly changed. This may have been related to the natural sedimentation of particulate matter. Our DGGE results showed that the number of recognized bacterial DGGE bands varied with time during disturbance (LID or HID), and significantly increased as the intensity of disturbance increased (Fig. S1). There is a plausible explanation for this phenomenon. In large shallow lakes, such as Lake Taihu, wind-induced water column mixing frequently disturbs the water-sediment interface (41). In these conditions, the upper layer of sediment is continuously re-suspended in the water column. The fluxes of organic matter between the sediment and the water column are presumed to be high, and sediment particles may be effectively transported into the overlying water. These organic particles harbor numerous bacteria (37), and re-suspension may enhance remineralization and induce dissolved nutrient and particulate organic matter fluxes into the water column, which can be used by bacteria (3, 25, 36). Previously, an increase in bacterial abundance and biovolume due to sediment re-suspension has been shown both in field and experimental studies (27, 35). However, the DGGE profiles of PABCC in C and D treatments were distinct even at the beginning of the experiment (Fig. S1). This might have been because they come from two different sampling sites. During the experiment, the slight changes in the number of recognized bacterial DGGE bands of C treatment might have been due to natural sedimentation of the particles, consistent with the slight decrease in the concentrations of nutrients.

There is a potential reason for the low resilience of the PABCC following two frequent disturbances by wind-waves. A combination of several hydrodynamic processes, acting on different spatial or temporal scales, drives the dynamics of aquatic ecosystems (18). Harris (11) described the response of microbial populations to disturbance events as a hierarchical effect. The extent of wind-wave disturbance affecting the microbial community is determined by both the force and duration of the disturbance. If the physical event is intense but of short duration (*e.g.* storms or typhoons), the microbial response occurs at a physiological level. If, however, the physical event is intense and of longer duration, it would be expected to see changes in growth rates and species composition (24). Our results also showed intensive changes in the abundance and composition of the particle-attached bacterial community in response to frequent wind-wave disturbances (Fig. 2; Table 2). In Lake Taihu, different wave processes occur very frequently due to its shallowness and particular shape, disturbing the water-sediment interface, and often causing intensive sediment re-suspension (41). In a study of wind speeds at the lake, daily maximum wind speeds (calculated as hourly average wind speed) higher than 5 m s^−1^ occurred 89.5% of the time, and wind speeds higher than 8 m s^−1^ occurred 34.2% of the time during the three-year period, 1997–1999 (9). Therefore, for the shallow and well-mixed Lake Taihu, with intermittent wind-induced wave disturbance, particle-attached bacterial communities were continuously changing and the resilience of the PABCC may be very low. Our results also bring us closer to previous reports that water turbidity caused by wind-wave disturbance was a significant environmental variable correlated with spatial variations in the bacterial community composition of the water column in Lake Taihu (38).

Although the PABCC in disturbed treatment achieved a degree of recovery by 4 days post-disturbance (Table 1), LIBSHUFF analysis showed that the composition of these communities on day 10 was still slightly different from the PABCC on day 0, and the composition of these communities on day 20 was significantly different from the PABCC on day 0 (Table 3). This result showed that the recovery was not complete. However, the recovery of PABCC following HID was significantly lower than that of LID (Table 1; Fig. 3), which suggests that, following frequent disturbance, disturbed particle-attached bacterial communities cannot recover or recovery is very slow. It is possible that after the disturbance had stopped, 4 days was insufficient for the bacterial community to return to its starting condition; more time for recovery may have been needed. However, more probable is that strong turbulence in the water column caused by the continuous disturbance had severely damaged the structure of particle-attached bacterial communities and the ecological niche had changed so much that a return to pre-disturbance levels was not possible. A study by Costerton *et al.* (5) also pointed out that microbial communities in natural environments subject to succession and fluctuation over time, recovery of most microbial communities needs not only natural nutrients, but also an ecological niche. Furthermore, most microbes transported away from their original habitat will atrophy, and the smallest change can cause loss of both vitality and niche; thus, recovery of microbial communities, if possible, requires a long period of time and, in some cases, recovery is not possible (15, 32). Therefore, following frequent disturbance, particle-attached bacterial communities cannot recover or the recovery is extremely slow.

The advective entrainment of surficial sediments into the water column by wind events generally occurs over time scales of hours to days (4, 17). Under these conditions, the fluxes of organic matter between sediment and water column are presumably high, and nutrients may be effectively transported into the water column (16). However, the upper layer of the sediment is continuously re-suspended into the water column, the flux of sediment bacterium on sediment particles is not a rare event, and the pelagic bacterial community may be a mixture of species primarily from the sediment and water column (16). Therefore, the contribution of sediment bacteria to the altered communities was important to understand changes in the PABCC following wind-wave disturbance. Unfortunately, we have no data on biomass, productivity of the sediment, or fluxes of bacteria between the sediment and the overlying water.

In summary, our study showed that wind-wave disturbance has an influence on the diversity and structure of particle-attached bacterial communities in a shallow lake. We observed the low resistance and low resilience of particle-attached bacterial communities following frequent wind-wave disturbances.

## Supplementary Information



## Figures and Tables

**Table 1:** Resistance and recovery of particle-attached bacterial communities for the control and disturbed treatments. LID, low-intensity disturbance; HID, high-intensity disturbance.

		Resistance: Minimum similarity to Day 0	Resistance: Average similarity to Day 0	Recovery: Day 10 (20) similarity to Day 0
Treatment D	LID	46.5	53.5	60.5
	HID	25.4	31.7	38.7
Treatment C		89.6	93.4	85.4

**Table 2:** Comparison of diversity estimators and coverage for bacterial 16S rRNA gene clone libraries constructed from 3 samples recovered from the control and disturbed treatments. D0, number of library samples in D treatment at day 0; D10, number of library samples in D treatment at day 10; D20, number of library samples in D treatment at day 20.

Sample	Clones	OTUs	Chao1 estimate	Shannon index (*H*′)	Coverage (%)	Simpson index
D0	85	21	32	2.42	89.4	0.12
D10	85	50	109.5	3.64	63.5	0.03
D20	85	31	114.3	2.62	76.5	0.11

**Table 3:** LIBSHUFF comparisons of the homology and heterogeneity of three clone libraries. Libraries were considered significantly different when *p*<0.0043, see Singleton *et al.* (2001) for details.

Comparison	*p*-value	Significantly different
D0 vs. D10		No
XY	0.0056	
YX	0.0072	
D0 vs. D20		Yes
XY	0.0032	
YX	0.0018	
D20 vs. D10		Yes
XY	0.0010	
YX	0.0020	
