# Low Resilience of the Particle-Attached Bacterial Community in Response to Frequent Wind-Wave Disturbance in Freshwater Mesocosms

**DOI:** 10.1264/jsme2.ME13032e

**Published:** 2014-03

KEQIANG SHAO^1^, GUANG GAO^1^, XIANGMING TANG^1^, YONGPING WANG^3^, LEI ZHANG^1,2^, BOQIANG QIN^1^

^1^*State Key Laboratory of Lake Science and Environment, Nanjing Institute of Geography and Limnology, Chinese Academy of Sciences, 73 Beijing East Road, Nanjing 210008, China;* ^2^*Graduate School of Chinese Academy of Sciences, Beijing 100039, China; and* ^3^*State Key Laboratory of Hydrology-Water Resources and Hydraulic Engineering, Nanjing, 223 Guangzhou Road, Nanjing 210029, China*

Volume 28, no. 4, Page 450–456, 2013

Page 450

Date of “Published online” is incorrect. It should be corrected as follows:

incorrect: November 26, 2013

correct: December 13, 2013

This correction has been completed on the electronic file of the present paper available at J-STAGE.

The editorial board expresses sincere apology for the misprinting.

Microbes and Environments editorial board

