# Supplementary material for: Low Resilience of the Particle-Attached Bacterial Community in Response to Frequent Wind-Wave Disturbance in Freshwater Mesocosms
Source: Microbes Environ. 2013 Dec 13;28(4):450–6. doi: 10.1264/jsme2.ME13032 (PMC4070706; doi:10.1264/jsme2.ME13032)

Supplemental material

Fig.S1. Denaturing gradient gel electrophoresis profiles of the particle-attached bacterial community from different samples in the disturbed treatment (D) and untreated control (C), LID, low-intensity disturbance; HID, high-intensity disturbance.

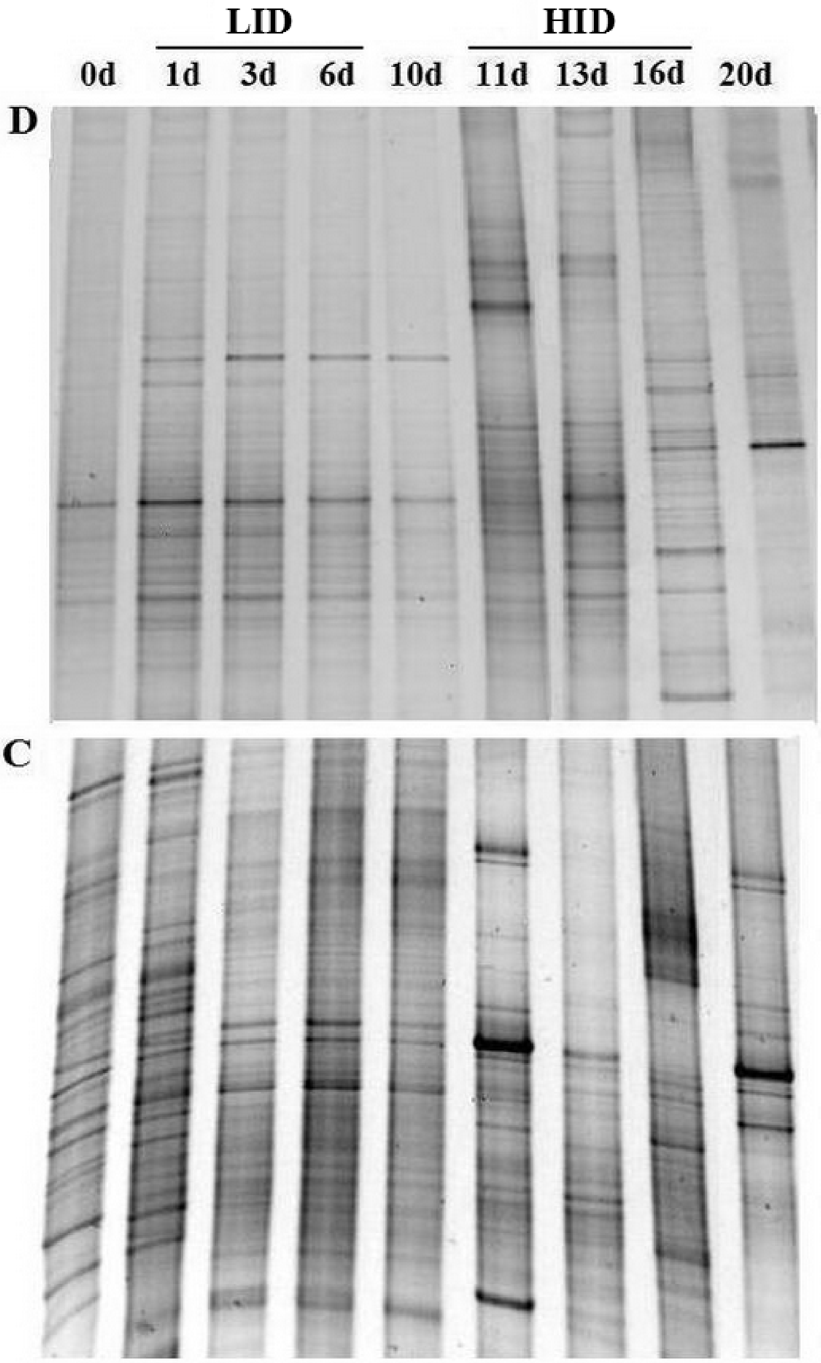

Supplement: Supplementary file 1 [file 28_450_s1.pdf]
